# Supplementary material for: A general framework to support cost-efficient fecal egg count methods and study design choices for large-scale STH deworming programs–monitoring of therapeutic drug efficacy as a case study
Source: PLoS Negl Trop Dis. 2023 May 17;17(5):e0011071. doi: 10.1371/journal.pntd.0011071 (PMC10228800; doi:10.1371/journal.pntd.0011071)
Supplement: S1 Info — (PDF) [file pntd.0011071.s001.pdf]

Supplementary Info S1. Summary of the standard operating procedures to time the preparatory steps and the egg counting for three FEC methods.

The detailed standard operating procedures (SOPs) to time the preparatory steps and the egg counting are for duplicate KK, for Mini-FLOTAC and for FECPAK^{G2} are described elsewhere (see S3-5 info of Vlaminck *et al*. [1]). In the sections below, we provide a summary of these SOPs for each fecal egg count method (FEC).

Kato-Katz

For each stool sample, two Kato-Katz (KK) thick smears were prepared according to the SOP (SOP; S3 info of [1]). The steps that needed timing were marked on the SOP. Timers were started when technicians took the first sample of each batch to prepare two thick smears. Timing of the preparation phase stopped when the final thick smear of the last sample of the batch was prepared. The number of samples per batch and the time to prepare duplicate KK thick smears for each of the samples were registered on record form 01 (S12 info of [1]). Preparation of batches with less than five samples was not timed. Since the trial setup did not allow for timing of the preparation of a single KK thick smear separately, we assume here that the time required for a single slide is half that required for a duplicate KK. Laboratory technicians measured the time it took them to read each individual slide. Once a slide was read, the timer was stopped and both the FECs as well as the reading time was recorded on record form 02 (S12 info of [1]). The time needed to read slide A was used to estimate the reading time for a single KK (as a function of the total egg count; see main paper). For duplicate KK, the reading time of slide A and B was combined. To calculate the FECs (expressed as eggs per gram of stool; EPG) of the different STH in a sample, total egg counts were multiplied by 24 for single KK and by 12 for duplicate KK.

Mini-FLOTAC

Samples were processed by the Mini-FLOTAC method according to the SOP as reported previously (S4 info of [1]). Per batch of samples, technicians recorded the time it took to prepare Mini-FLOTAC devices, along with the number of samples contained within that batch, using record form 04 (S12 info of [1]). Preparation of batches with less than five samples was

not timed. Laboratory technicians measured the time it took to count the STH eggs present in both mini-FLOTAC chambers. After examination, egg counts and the reading time were recorded on record form 05 (S12 info of [1]). To calculate the FECs (in EPG), egg counts were multiplied by 10.

FECPAK^{G2}

The FECPAK^{G2} method was performed according to the SOP as previously described (S5 info of [1]). Briefly, preparing the stool samples for analysis by the Micro-I digital microscope system consisted of three different steps. First, stool was homogenized in tap water within a Fill-FLOTAC device, after which it was transferred into a FECPAK^{G2} sedimenter to allow STH eggs to sediment. After overnight sedimentation, the supernatant was poured off and saturated saline solution (specific density = 1.2) was added to the remaining slurry. The whole content of the sedimenter was then poured into a FECPAK^{G2} filtration unit from which two separate aliquots were taken and transferred to two wells of a FECPAK^{G2} cassette. After this, cassettes were placed on a horizontal surface for at least 20 minutes to allow accumulation of STH eggs at the tip of the rod inside the two cassette wells (accumulation step). Finally, cassettes were placed in the Micro-I device for image capture. The device automatically imaged both wells and stored the images prior to uploading them to the FECPAK^{G2} server. For each batch of samples containing more than five samples, technicians measured the time needed to (i) perform the stool homogenization, (ii) fill the cassettes and to (iii) have the cassettes read by the Micro-I device. These timing measurements were recorded on record form 07 (S12 info of [1]). To count the number of STH eggs in the sample, laboratory technicians identified and counted any STH egg present in the images using specialized software. Results of this mark-up were saved automatically for reporting and analysis. The total number of eggs counted in both wells were multiplied by 34 to calculate the FECs. The time it took to mark-up the STH eggs on the images of each sample was noted on record form 08 (S12 info of [1]).

References

1. Vlaminck J, Cools P, Albonico M, Ame S, Ayana M, Bethony J, et al. Comprehensive evaluation of stool-based diagnostic methods and benzimidazole resistance markers to assess drug efficacy and detect the emergence of anthelmintic resistance: A Starworms study protocol. PLoS Negl Trop Dis. 2018 Nov 2;12(11):e0006912. doi: 10.1371/journal.pntd.0006912.
