# Supplementary material for: A general framework to support cost-efficient fecal egg count methods and study design choices for large-scale STH deworming programs–monitoring of therapeutic drug efficacy as a case study
Source: PLoS Negl Trop Dis. 2023 May 17;17(5):e0011071. doi: 10.1371/journal.pntd.0011071 (PMC10228800; doi:10.1371/journal.pntd.0011071)
Supplement: S2 Info — (PDF) [file pntd.0011071.s002.pdf]

Supplementary Info S2. Summary of the standard operating procedures to time the data entry.

Study data recorded on paper were entered into an Excel file in duplicate. Demographic data collected at baseline from each study participant were recorded on log form 01 (see S12 info of Vlaminck *et al*. [1]). The demographic data consisted of subject ID, school ID, grade, age and sex of the participant, date of recruitment and treatment. This information was later digitized in an Excel file (see S13 info of Vlaminck *et al*. [1]). The number of participants for which data were entered and the time it took to perform this task were registered on the timing record form 01 (see S12 info of Vlaminck *et al*. [1]). For FECPAK^{G2}, these data were registered in the specialized FECPAK^{G2} software and timed separately on record form 07 (see S12 info of Vlaminck *et al*. [1]). The total time of each data entry session was divided by the number of individuals for which demographic data was entered to obtain an average time needed to enter data for one participant.

At regular intervals, FEC data recorded on paper were entered into an Excel file (see S13 info of Vlaminck *et al*. [1]). Digitization of all duplicate Kato-Katz (KK) thick smear fecal egg counts (FECs; 6 integer entries = 3 STH x 2 slides) was timed and registered on timing record form 02 (see S12 info of Vlaminck *et al*. [1]) and later entered in a separate Excel file (see S14 info of Vlaminck *et al*. [1]). Digitization of FEC data obtained with Mini-FLOTAC was not timed. Instead, the time needed for FEC data entry of Mini-FLOTAC was estimated to be half of the time needed to enter FEC data obtained by duplicate KK data (6 entries: 3 STHs x 2 slides) as there were only half as many entries to be made (3 STH x 1 mini-FLOTAC).

References

1. Vlaminck J, Cools P, Albonico M, Ame S, Ayana M, Bethony J, et al. Comprehensive evaluation of stool-based diagnostic methods and benzimidazole resistance markers to assess drug efficacy and detect the emergence of anthelmintic resistance: A Starworms study protocol. PLoS Negl Trop Dis. 2018 Nov 2;12(11):e0006912. doi: 10.1371/journal.pntd.0006912.
