# Supplementary material for: A general framework to support cost-efficient fecal egg count methods and study design choices for large-scale STH deworming programs–monitoring of therapeutic drug efficacy as a case study
Source: PLoS Negl Trop Dis. 2023 May 17;17(5):e0011071. doi: 10.1371/journal.pntd.0011071 (PMC10228800; doi:10.1371/journal.pntd.0011071)
Supplement: S4 Info — (PDF) [file pntd.0011071.s004.pdf]

S4 Info. Methods to estimate the FECs of Mini-FLOTAC and FECPAK^{G2} based on duplicate Kato-Katz.

Statistical data analysis

To parameterize simulations for EPG results based on the four different diagnostic approach (single Kato-Katz (KK), duplicate KK, mini-FLOTAC and FECPAK^{G2}), we first quantified the association between the four tests using a generalized linear mixed model (GLMM) for egg counts:

$$\text{count}_{ijt} \sim \text{NegativeBinomial}(\mu = \hat{y}_{ijt}, shape = k_t) \text{ or } \text{count}_{it} \sim \text{Poisson}(\mu = \hat{y}_{it})$$

$$\log(\hat{y}_{ijt}) = \beta_{0ij} + \beta_1 \cdot I(\text{KK1}) + \beta_2 \cdot I(\text{MF}) + \beta_3 \cdot I(\text{FP}) + \log(\text{weight}_t)$$

$$\beta_{0ij} \sim \Gamma(k_j, k_j/\mu_j)$$

This GLMM describes four repeated egg counts per individual, where the expected egg count $\hat{y}_{ijt}$ with test *t* in individual *i* in school *j* is assumed to follow either a negative binomial or Poisson distribution. The expected egg count was determined by a random intercept per individual (representing the expected eggs per gram of stool (EPG) based on duplicate KK), a coefficient for the type of test (single KK, MF, or FP; duplicate KK was the reference), and an offset representing the amount of stool tested (in grams). Here, $I(\text{X})$ is an indicator function that equals 1 if count_{ijt} is based on test X, and 0 otherwise. We used the natural logarithm as the link function, such that coefficients represent the logarithm of the relative difference in expected EPGs between the diagnostic test types.

If the Bayesian 95%-credible interval of any of the coefficients $\beta_{1:3}$ included zero, this coefficient was dropped from the model. Further, for each test, the choice of modelling counts as a negative binomial versus Poisson distribution was based on whether or not the test-specific overdispersion parameter k_t converged to a value <10 (high values of k_t indicate a more Poisson-like distribution).

The GLMM was implemented in a Bayesian framework and parameters were estimated with Hamiltonian Monte Carlo, using the package *rstan* [1] in R [2]. Priors for parameters were either weakly informative ($k_t \sim N^{+}(0,5)$ and $\beta_{1:3} \sim N(0,1)$) or flat improper priors (school-level mean epg μ_j and overdispersion k_j). The posterior means of $\exp(\beta_{1:3})$ were used in the simulation framework to translate between expected EPGs based on different diagnostic tests.

Tables S4.1 and **S4.2** summarize the results of the statistical data analysis.

Table S4.1. The egg recovery relative to duplicate Kato-Katz thick smear

**Species**	**Egg recovery relative to double Kato-Katz**		
	Single Kato-Katz	Mini-FLOTAC	FECPAK
A. lumbricoides	1.00 (1.00-1.00) *	0.65 (0.56-0.74)	0.25 (0.21-0.29)
T. trichuria	1.00 (1.00-1.00) *	1.01 (0.94-1.08)	0.71 (0.57-0.87)
Hookworm	1.00 (1.00-1.00) *	0.8 (0.73-0.88)	0.57 (0.48-0.67)

* Egg counts based on a single Kato-Katz slide showed no evidence of a systematic difference with average counts based on duplicate Kato-Katz and were therefore assumed to have the same expectation in terms of eggs per gram stool as duplicate Kato-Katz results.

Table S4.2. The overdispersion of egg counts (shape parameter negative binomial distribution) for Mini-FLOTAC and FECPAK^{G2}. Egg counts from repeated Kato-Katz based on the same stool sample showed no evidence of overdispersion and were therefore assumed to follow a Poisson distribution.

**Species**	**Overdispersion of egg counts (shape parameter)**	
	Mini-FLOTAC	FECPAK
A. lumbricoides	0.58 (0.49-0.68)	0.52 (0.43-0.62)
T. trichuria	3.02 (2.51-3.58)	0.71 (0.57-0.87)
Hookworm	1.47 (1.23-1.73)	0.57 (0.48-0.69)

References

1. Stan Development Team (2022). RStan: the R interface to Stan. R package version 2.21.5. https://mc-stan.org/.
2. R Core Team (2022). R: A language and environment for statistical computing. R Foundation for Statistical Computing, Vienna, Austria. URL https://www.R-project.org/.
