# Supplementary material for: A general framework to support cost-efficient fecal egg count methods and study design choices for large-scale STH deworming programs–monitoring of therapeutic drug efficacy as a case study
Source: PLoS Negl Trop Dis. 2023 May 17;17(5):e0011071. doi: 10.1371/journal.pntd.0011071 (PMC10228800; doi:10.1371/journal.pntd.0011071)
Supplement: S5 Info — (PDF) [file pntd.0011071.s005.pdf]

S5 Info. Detailed description and parameterization of the simulation model.

General simulation framework

As described in the main text, we adapted the simulation framework described by Coffeng and colleagues [1], accounting for the following sources of variation in egg counts:

1. Inter-individual variability in mean egg intensity due to variation in infection levels between individuals (assumed to follow a gamma distribution);
2. Day-to-day variability in mean egg intensity within an individual due to heterogeneous egg excretion over time (assumed to follow a gamma distribution);
3. Variability in egg counts between repeated aliquots of a stool sample due to the aggregated distribution of eggs in faeces (assumed to follow a Poisson or a gamma-Poisson (i.e., negative binomial) distribution);
4. Inter-individual variability in the effect of drug treatment in terms of the ERR (assumed to follow a beta distribution).

For the quantification of each gamma distribution, we follow the approach of Denwood et al [2] in using the coefficient of variation (cv) as a standardised measure of variability, which is related to the shape parameter k of a gamma distribution by taking $k = cv^{-2}$.
Baseline egg counts for a particular STH species were simulated as follows:

$$\mu_i \sim \Gamma\left(k_i, \frac{k_i}{\mu}\right) \qquad \text{Eq. (1)}$$

$$\mu_{id} \sim \Gamma\left(k_d, \frac{k_d}{\mu_i}\right) \qquad \text{Eq. (2)}$$

$$\mu_{ids}\begin{cases}\sim\Gamma\left(k_s\cdot w_s,\dfrac{k_s}{\mu_{id}\cdot\rho_s}\right) & \text{if } cv_S>0\\ =\mu_{id}\cdot w_s\cdot\rho_s & \text{if } cv_S=0\end{cases} \quad \text{Eq. (3)}$$

$$\text{baseline count}_{ids}\sim Pois(\mu_{ids}) \quad \text{Eq. (4)}$$

Here, μ represents the average baseline fecal egg count (FEC; expressed in eggs per gram of stool (EPG)) at the population level, μ_i is the expected FEC for an individual i, μ_{id} is the expected FEC on a particular day d in individual i, and μ_{ids} is the expected egg count in an aliquot of stool for FEC method s. The latter depends on the weight w_s of the aliquot of stool sample used and the relative performance ρ_s of FEC method s in recovering eggs from stool, compared to Kato-Katz thick smear (KK; $\rho_{KK}=1$). The term ρ_s was included to capture that Mini-FLOTAC and FECPAK^{G2} yield systematically lower FEC estimates than KK [3]. Gamma distributions are indicated by Γ, parameterised in terms of shape k and rate $\frac{k}{\mu}$; $Pois$ indicates the Poisson distribution. Higher values of k indicate a lower coefficient of variation and therefore less over-dispersion of egg counts. Species-specific values of k_i and k_d (variability between individuals and within individuals over time) were estimated based on data from clinical trials during which a duplicate KK was performed on two consecutive stool samples both at baseline and follow-up [4,5]. Species and FEC method-specific values of k_s (variability between repeated aliquots of the same stool sample) and ρ_s (egg recovery performance) were estimated from the egg count data [3] (see **S4 Info** for more details).

Post-treatment egg counts were generated using a similar process as for the baseline counts, using an individual's expected baseline EPG μ_i scaled by $1-r_i$ (r for reduction), which is the true drug efficacy in individual i:

$$r_i\sim Beta(\alpha_{\mathrm{ERR}},\beta_{\mathrm{ERR}}) \quad \text{Eq. (5)}$$

$$v_{id}\sim\Gamma\left(k_d,\frac{k_d}{\mu_i\cdot(1-r_i)}\right) \quad \text{Eq. (6)}$$

$$v_{ids} \begin{cases} \sim \Gamma\left(k_s \cdot w_s, \dfrac{k_s}{v_{id} \cdot \rho_s}\right) & \text{if } cv_S > 0 \\ = v_{id} \cdot w_s \cdot \rho_s & \text{if } cv_S = 0 \end{cases} \qquad \text{Eq. (7)}$$

$$\text{follow-up count}_{ids} \sim Pois(v_{ids}) \qquad \text{Eq. (8)}$$

Here, $Beta$ is the beta distribution of variability in treatment effects between individuals i with shape parameters α_{ERR} and β_{ERR}. Values of these shape parameters were chosen such that the mean efficacy (ERR; $\mu_{\text{ERR}} = \alpha_{\text{ERR}}/(\alpha_{\text{ERR}} + \beta_{\text{ERR}})$) equaled a value 5%-points under the species-specific threshold for reduced efficacy and the 2.5th and 97.5th percentiles for inter-individual variability spanned 20%-points, centered approximately around the mean. A full overview of all parameter values by species and diagnostic technique is provided in **Table 1** in the main text.

References

1. Coffeng LE, Malizia V, Vegvari C, Cools P, Halliday KE, Levecke B, et al. Impact of Different Sampling Schemes for Decision Making in Soil-Transmitted Helminthiasis Control Programs. J Infect Dis. 2020 Jun 11;221(Suppl 5):S531-S538. doi: 10.1093/infdis/jiz535. PMID: 31829425; PMCID: PMC7289558.
2. Denwood MJ, Love S, Innocent GT, Matthews L, McKendrick IJ, Hillary N, Smith A, Reid SW. Quantifying the sources of variability in equine faecal egg counts: implications for improving the utility of the method. Vet Parasitol. 2012 Aug 13;188(1-2):120-6. doi: 10.1016/j.vetpar.2012.03.005.
3. Cools P, Vlaminck J, Albonico M, Ame S, Ayana M, José Antonio BP, et al. Diagnostic performance of a single and duplicate Kato-Katz, Mini-FLOTAC, FECPAKG2 and qPCR for the detection and quantification of soil-transmitted helminths in three endemic countries. PLoS Negl Trop Dis. 2019 Aug 1;13(8):e0007446. doi: 10.1371/journal.pntd.0007446.
4. Knopp S, Mohammed KA, Speich B, Hattendorf J, Khamis IS, Khamis AN, et al. Albendazole and mebendazole administered alone or in combination with ivermectin against *Trichuris trichiura*: a randomized controlled trial. Clin Infect Dis. 2010;51(12):1420-8.
5. Steinmann P, Utzinger J, Du ZW, Jiang JY, Chen JX, Hattendorf J, Zhou H, Zhou XN. Efficacy of single-dose and triple-dose albendazole and mebendazole against soil-transmitted helminths and Taenia spp.: a randomized controlled trial. PLoS One. 2011;6(9):e25003. doi: 10.1371/journal.pone.0025003.
