# Supplementary material for: A general framework to support cost-efficient fecal egg count methods and study design choices for large-scale STH deworming programs–monitoring of therapeutic drug efficacy as a case study
Source: PLoS Negl Trop Dis. 2023 May 17;17(5):e0011071. doi: 10.1371/journal.pntd.0011071 (PMC10228800; doi:10.1371/journal.pntd.0011071)
Supplement: S6 Info — (PDF) [file pntd.0011071.s006.pdf]

S6 Info. The calculation of the total operational costs to monitor drug efficacy

As described in the main manuscript, for each simulated survey, we calculated the operational costs in terms of (i) the cost of consumables to collect and process samples, (ii) the salary for a single mobile field team that comprised of one nurse and three laboratory technicians, and (iii) the cost of transport, including car rental, salary of the driver and gasoline:

$$cost_{total} = cost_{consumables} + cost_{personnel} + cost_{transport} \quad \text{Eq. (1)}$$

In the next three sub-sections we describe the cost of consumables, salary, and transport in detail.

Consumable costs

The consumable costs for a simulated survey design based on $a \times b$ baseline egg counts (i.e., a stool samples per person, each tested with b aliquots) and $c \times d$ follow-up counts were defined as:

$$cost_{consumables} = N_{baseline} \times a \times \left[cost_{sample} + b \times cost_{aliquot,b}\right] + N_{follow-up} \times c \times \left[cost_{sample} + d \times cost_{aliquot,d}\right] \quad \text{Eq. (2)}$$

Here, $cost_{sample}$ represents the consumable cost of processing a single stool sample and $cost_{aliquot,X}$ is the cost per aliquot when processing a set of X aliquots from the same stool sample (because testing multiple aliquots costs less per aliquot). The number of tested stool samples at baseline ($N_{baseline}$) and follow-up ($N_{follow-up}$) depend on the initial number N_{ind} of tested individuals and, depending on the survey design, the number of individuals N_{pos} that test positively on their first baseline stool sample:

$$N_{baseline} = \begin{cases} N_{ind} + N_{pos} & \text{if survey design is } SSR \\ N_{ind} & \text{otherwise} \end{cases} \quad \text{Eq. (3)}$$

$$N_{follow-up} = \begin{cases} N_{pos} & \text{if survey design is } SSR \text{ or } SS \\ N_{ind} & \text{otherwise} \end{cases} \quad \text{Eq. (4)}$$

For the sake of simplicity, we have omitted subscripts for the used FEC method in of the equations. See **Tables 2 and 3** in the main manuscript for an overview of how parameters vary by FEC method.

Personnel costs

The cost of a single mobile field team (including salary and lodging) was set to depend on the number of N_{days} it takes to complete the survey with N_{team} team members and the cost per person per day ($cost_{perdiem}$):

$$cost_{personnel} = N_{days} \times N_{team} \times cost_{perdiem} \qquad \text{Eq. (5)}$$

Here, the total number of days (N_{days}) required to complete the baseline and follow-up surveys depends on the total person-time needed to process and analyze N stool samples with X aliquots tested per sample and subsequently record the results ($T_{\text{total},N,X}$, in seconds), and the total number of person-time available to perform the work ($T_{\text{work/day}}$, in seconds per day):

$$N_{days} = N_{days,baseline} + N_{days,follow-up} \qquad \text{Eq. (6)}$$

$$N_{days,baseline} = \begin{cases} \left\lceil \frac{T_{\text{total},N_{ind},b}}{T_{\text{work/day}}} \right\rceil + \left\lceil \frac{T_{\text{total},N_{pos},b}}{T_{\text{work/day}}} \right\rceil & \text{if survey design is } SSR \\ \left\lceil \frac{T_{\text{total},N_{ind},b}}{T_{\text{work/day}}} \right\rceil & \text{otherwise} \end{cases}$$

$$N_{days,follow-up} = \begin{cases} \left\lceil \frac{T_{\text{total},N_{pos},d}}{T_{\text{work/day}}} \right\rceil & \text{if survey design is } SSR \text{ or } SS \\ \left\lceil \frac{T_{\text{total},N_{ind},d}}{T_{\text{work/day}}} \right\rceil & \text{otherwise} \end{cases}$$

Here, the total person-time available per day to perform the work (the denominator) was defined as:

$$\mathrm{T}_{\mathrm{work/day}} = N_{technicians} \times 4\mathrm{h} \times 60\mathrm{min} \times 60\mathrm{sec} \quad \text{Eq. (7)}$$

The person-time required to perform the work ($T_{\mathrm{total},N,X}$, the numerator) was defined as a function of the number of tested stool samples (N) which, depending on the survey design, is either the number of initially screened individuals ($N = N_{ind}$), or the number of individuals that tested positive on their first baseline stool sample ($N = N_{pos}$). The total required person-time further depends the number of aliquots tested per sample (X), which can differ between the baseline ($X = b$) and follow-up surveys ($X = d$). For both the baseline and follow-up surveys (and a potential second baseline assessment in case of the SSR survey design), we round up to whole days, as indicated by the ceiling function "$\lceil \ldots \rceil$". This captures the notion that personnel have to be paid for the whole day, and that repeated stool samples are collected from individuals on different days.

The total person-time $T_{\mathrm{total},N,X}$ required to complete analysis of N stool samples consists of the time required to enter demographic data ($T_{\mathrm{demography}}$), the time required to prepare X aliquots per sample for egg counting ($T_{\mathrm{prep},X}$), the time required to count the eggs ($T_{\mathrm{count},N,X}$), and the time required to record the results ($T_{\mathrm{record},X}$):

$$T_{\mathrm{total},N,X} = N \times \left(T_{\mathrm{demography}} + T_{\mathrm{prep},X} + T_{\mathrm{record},X}\right) + T_{\mathrm{count},N,X} \quad \text{Eq. (8)}$$

All elements except the time to count eggs scale directly with the number of stool samples that are tested (N). In contrast, the time required to count eggs in X aliquots of N stool samples ($T_{\mathrm{count},N,X}$) depends on the number of eggs that are counted in each aliquot and a function $f(c)$ that returns the time required to count c eggs:

$$T_{\mathrm{count},N,X} = \sum_{i=1}^{N} \sum_{j=1}^{X} f(\mathrm{c}_{ij}) \quad \text{Eq. (9)}$$

Here we assume that the objective of the survey is to quantify the intensity of infection by counting all eggs in each aliquot, and that we are only considering one STH species at a time. Further note that, also here, we have omitted subscripts for the used FEC method in of the

equations. See **Tables 2 and 3** in the main manuscript for an overview of how parameters vary by FEC method.

Transport costs

The cost of transport depends on the number of survey days (Eq. 6) and a daily cost of car rental, salary of the driver and gasoline ($travel_{perdiem}$):

$$cost_{transport} = N_{days} \times travel_{perdiem} \qquad \text{Eq. (10)}$$

Cost of initial travel to and return of the study team from the study site was not considered, assuming that it is the same for all study designs and assuming that all study designs can be completed in a single study location.
