# Supplementary material for: A general framework to support cost-efficient fecal egg count methods and study design choices for large-scale STH deworming programs–monitoring of therapeutic drug efficacy as a case study
Source: PLoS Negl Trop Dis. 2023 May 17;17(5):e0011071. doi: 10.1371/journal.pntd.0011071 (PMC10228800; doi:10.1371/journal.pntd.0011071)
Supplement: S7 Info — (PDF) [file pntd.0011071.s007.pdf]

S7 Info. Detail information on the calculation of the time-to-result

Preparing samples for analysis

The preparation of samples was timed in batches of 5 to 10 samples for duplicate Kato-Katz (KK), Mini-FLOTAC and FECPAK^{G2}, separately (**Fig 1** of the main document). Across the four trial sites, the preparation of 2,159 duplicate KK in 243 different batches was timed. On average (standard deviation (SD)), each timed batch consisted of 8.9 (1.7) stool samples. The average (SD) preparation time for one duplicate KK was 135 sec (368). Since the set-up of our trial did not allow to time the preparation of a single KK slide separately, we divided the time it took to prepare a duplicate KK by two to reflect the time needed for a single KK preparation (= 67 sec (184)).

In total, the preparation of 2,133 Mini-FLOTACs in 240 different batches was timed. Batches consisted of an average (SD) of 8.9 (1.7) stool samples. On average (SD) 131 sec (36) were needed to prepare a single Mini-FLOTAC. As a second Mini-FLOTAC can be filled from the same Fill-FLOTAC (no need to weigh and homogenize the sample for a duplicate Mini-FLOTAC), we multiplied the median time required to process a single Mini-FLOTAC by 1.5 to estimate the median time for a duplicate Mini-FLOTAC (197 sec).

For FECPAK^{G2}, the preparation of 1,981 samples was timed across 221 different batches including on average (SD) 9.0 (1.6) stool samples. For each batch, three different manipulations were timed. First, the average time (SD) needed to prepare the samples in the FECPAK^{G2} sedimenters was estimated at 142 sec (45). Second, a mean of 174 sec (78) was needed to fill and prepare the FECPAK^{G2} cassette. Finally, it took on average 280 sec (45) to image one filled cassette in the Micro-I device. Combining all three preparation steps, the total average preparation time amounted to 596 sec for a single FECPAK^{G2}. To estimate the time for a

duplicate FECPAK^{G2}, we doubled the time for each of the different steps, except for the step to prepare the samples in the FECPAK^{G2} sedimenters, resulting in a total time of 1,050 sec (= 142 sec + 2 x 174 sec + 2 x 280 sec).

Counting eggs

The time it took to count the STH eggs was registered for 4,750 KK slides (Brazil: 568, Ethiopia: 1,496; Lao PDR: 1,478 and Tanzania: 1,208); 2,308 Mini-FLOTAC devices (Brazil: 284, Ethiopia: 748; Lao PDR: 671 and Tanzania: 605) and 2,256 FECPAK^{G2} images (Brazil: 272, Ethiopia: 742; Lao PDR: 644 and Tanzania: 598). On average (SD) it took a technician 413 sec (323) to visually detect and count STH eggs in a single KK slide. For Mini-FLOTAC and FECPAK^{G2} these values were 632 sec (536) and 185 sec (326), respectively. As illustrated in **Fig 3** of the main document**,** there was a linear association between the log transformed egg counts and the log transformed total time required to count all eggs, requiring a longer reading time when more STH eggs are to be counted.

Data entry

Demographic data were entered by eight data entry clerks (two in each of the four study sites) during a total of 102 different data entry sessions with demographic data being entered for 2,944 individuals (i.e. data of 1,472 individuals entered twice). The mean time (SD) needed to enter demographic data (7 parameters (see **Methods** section of the main document) for one study participant was 15 sec (9). We assume that the mean time needed for demographic data entry was the same for each FEC method for which it was required (single and duplicate KK, and Mini-FLOTAC). For FECPAK^{G2}, demographic data was entered using their specific software and the mean (SD) time equaled 34 sec (19) per individual.

Finally, we calculated the time needed to enter duplicate KK results for 3,834 samples during 151 data entry sessions. The mean (SD) number of duplicate KK FECs entered per data entry session was 25 (16). The mean time needed to enter one duplicate KK result was 18 (11) sec. For single KK and Mini-FLOTAC these values were divided by two as only three entries were needed instead of six. For the FECPAK^{G2} method, no FEC data entry was required as the software automatically registers and stores mark-up data.
