# Supplementary figures and images for: A general framework to support cost-efficient fecal egg count methods and study design choices for large-scale STH deworming programs–monitoring of therapeutic drug efficacy as a case study
Source: PLoS Negl Trop Dis. 2023 May 17;17(5):e0011071. doi: 10.1371/journal.pntd.0011071 (PMC10228800; doi:10.1371/journal.pntd.0011071)

*Ascaris*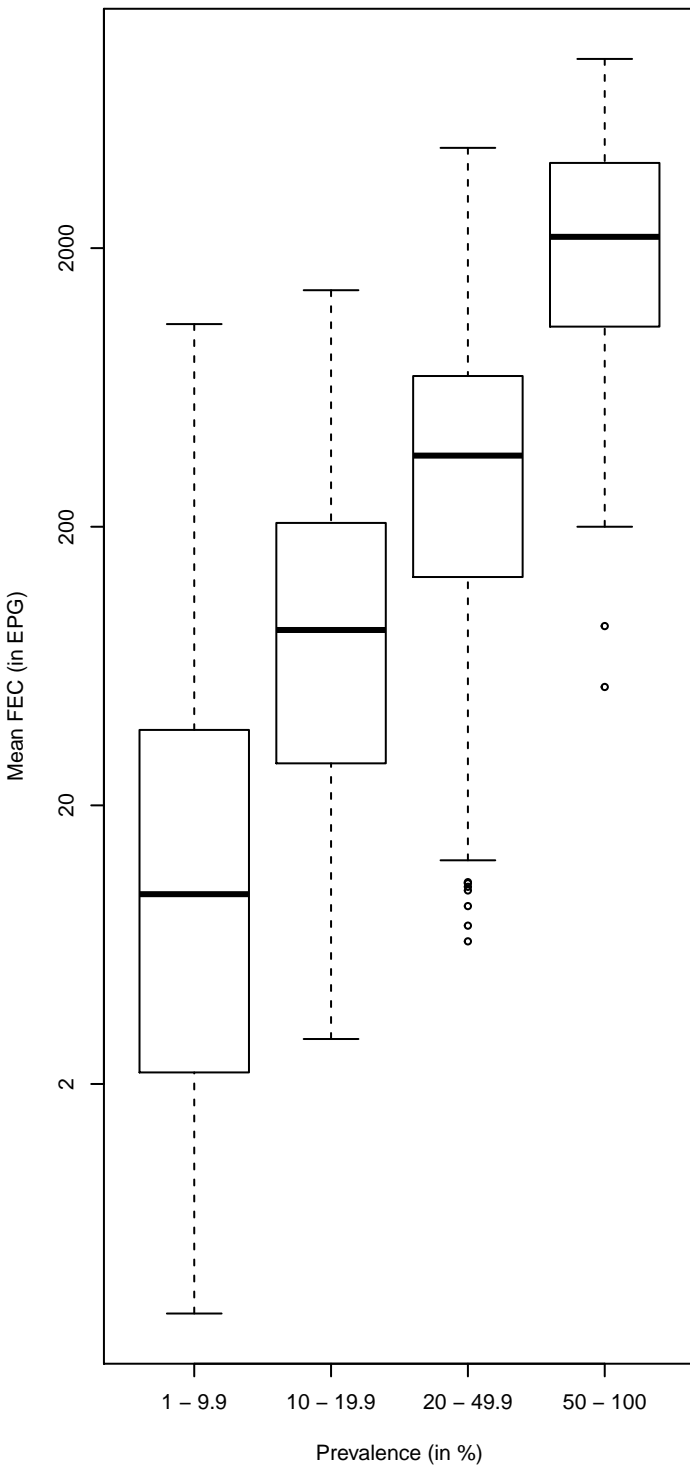*Trichuris*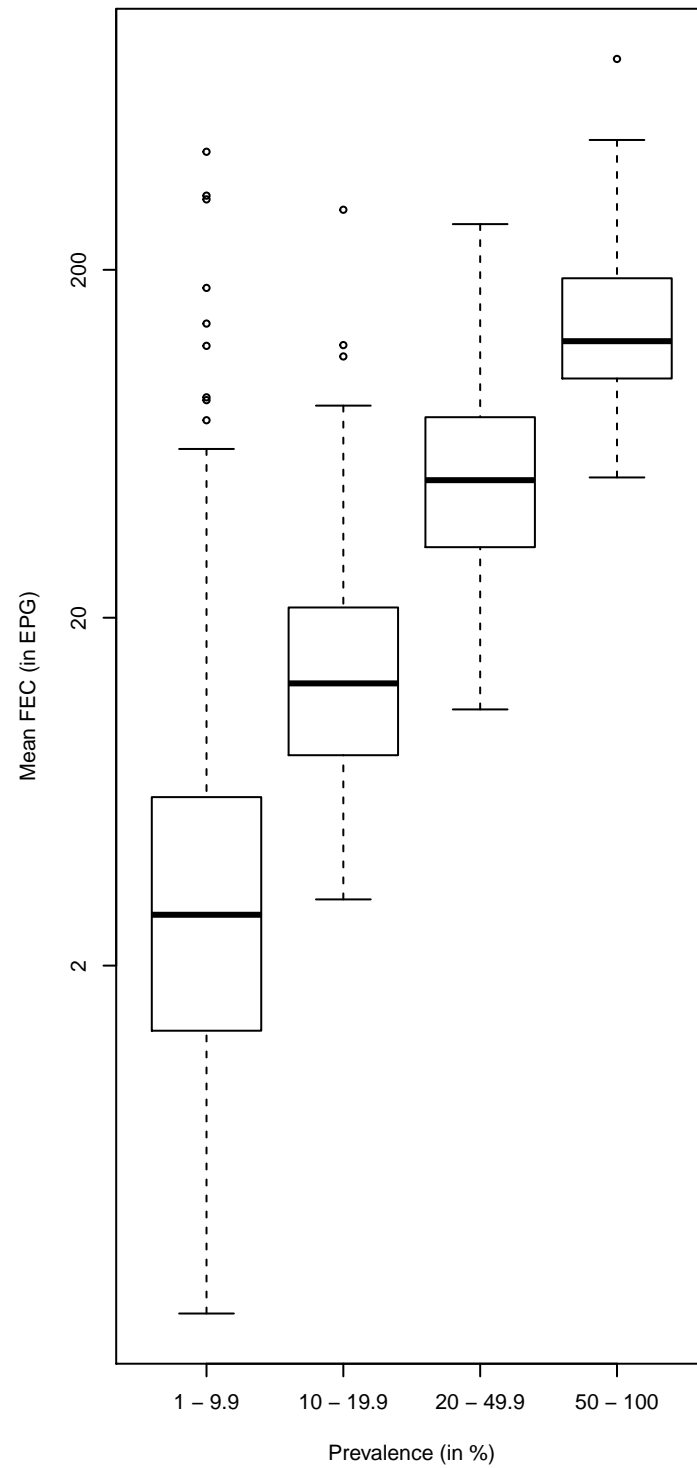**Hookworm**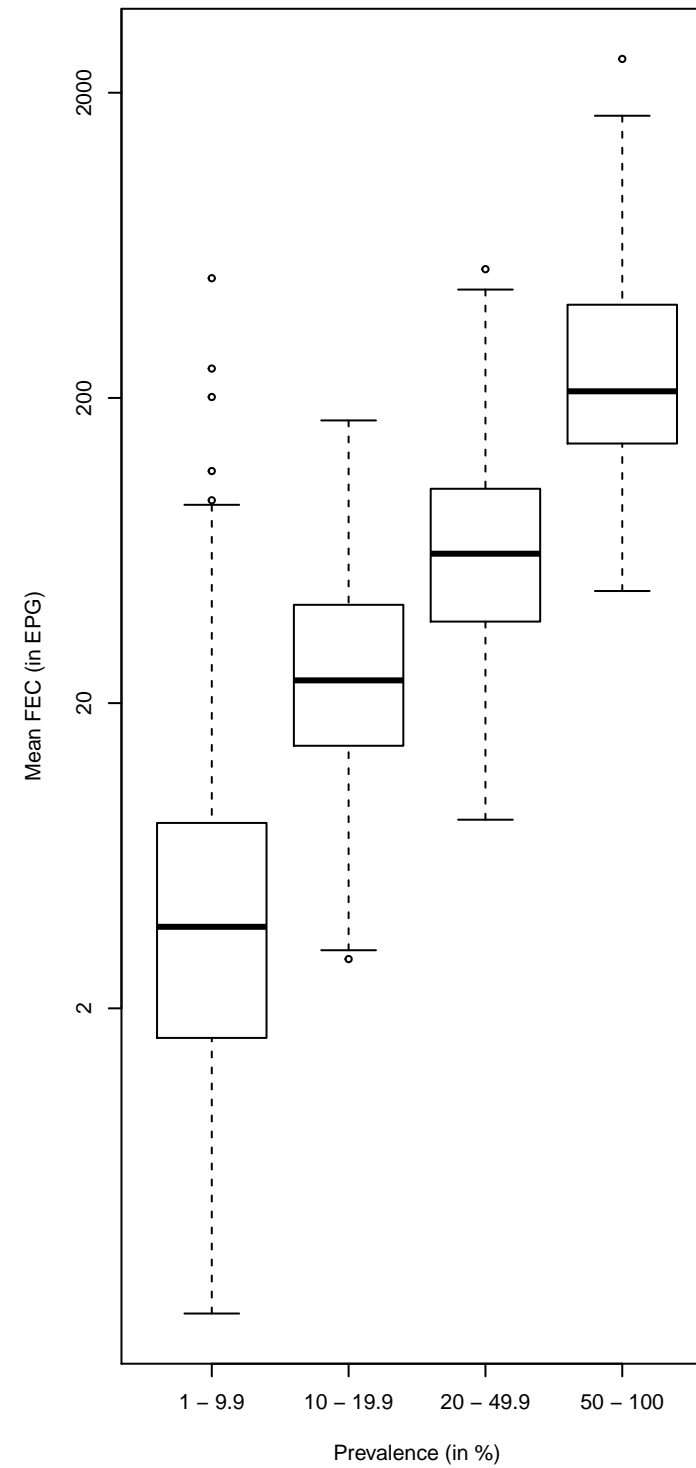

Supplement: S1 Fig — (PDF) [file pntd.0011071.s008.pdf]

A

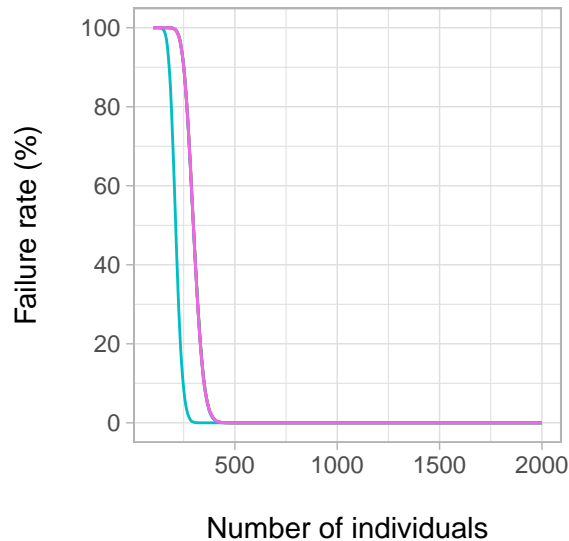

B

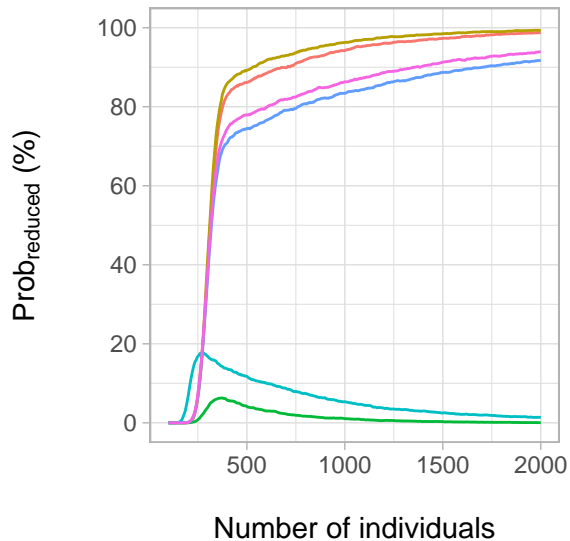

C

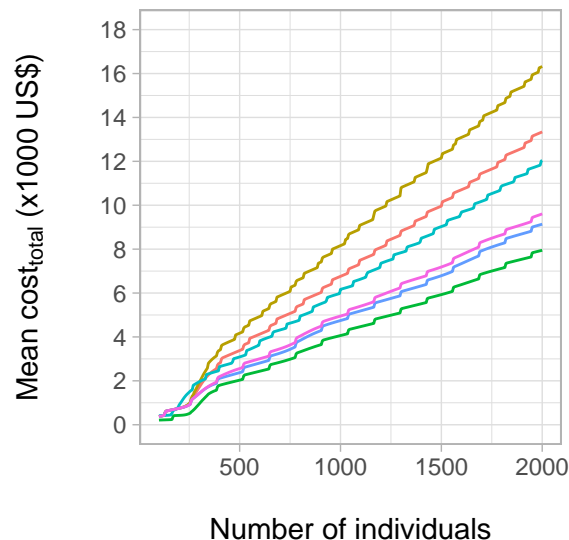

D

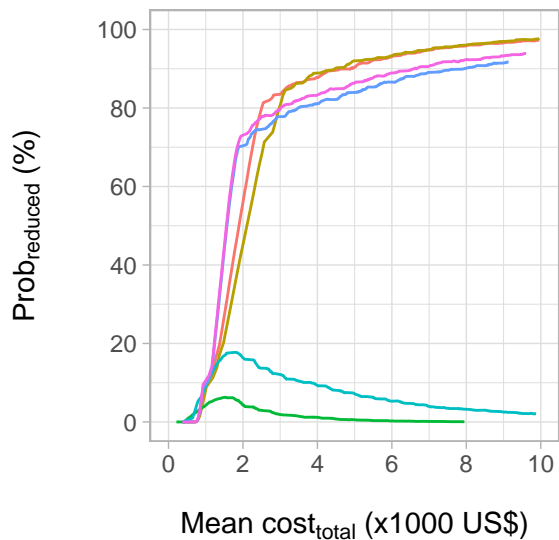

NS<sub>1x1/1x1</sub>   SS<sub>1x1/1x1</sub>   SSR<sub>1x1/1x1</sub>  
 NS<sub>1x1/1x2</sub>   SS<sub>1x2/1x2</sub>   SSR<sub>1x1/1x2</sub>

Supplement: S2 Fig — This figure shows the impact of the survey design and sample size on the failure rate (Panel A), probability of correctly detecting truly reduced efficacy (probreduced; Panel B) and the mean total survey cost (costtotal; Panel C). To gain more insights into the most cost-efficient survey design, the probability of correctly detecting reduced drug efficacy probreduced was plotted as a function of the mean costtotal (Panel D). For each of the four panels, we only consider the use of Kato-Katz in areas with low levels of Ascaris infection (mean FEC = 9.6 EPG). NS = no selection; SS = screen and select; SSR = screen, select, and retest. Note, for panel A, all survey designs other than SS1x2/1x2 are identical to SSR1x1/1x2. (PDF) [file pntd.0011071.s009.pdf]

Prob<sub>reduced</sub> (%)

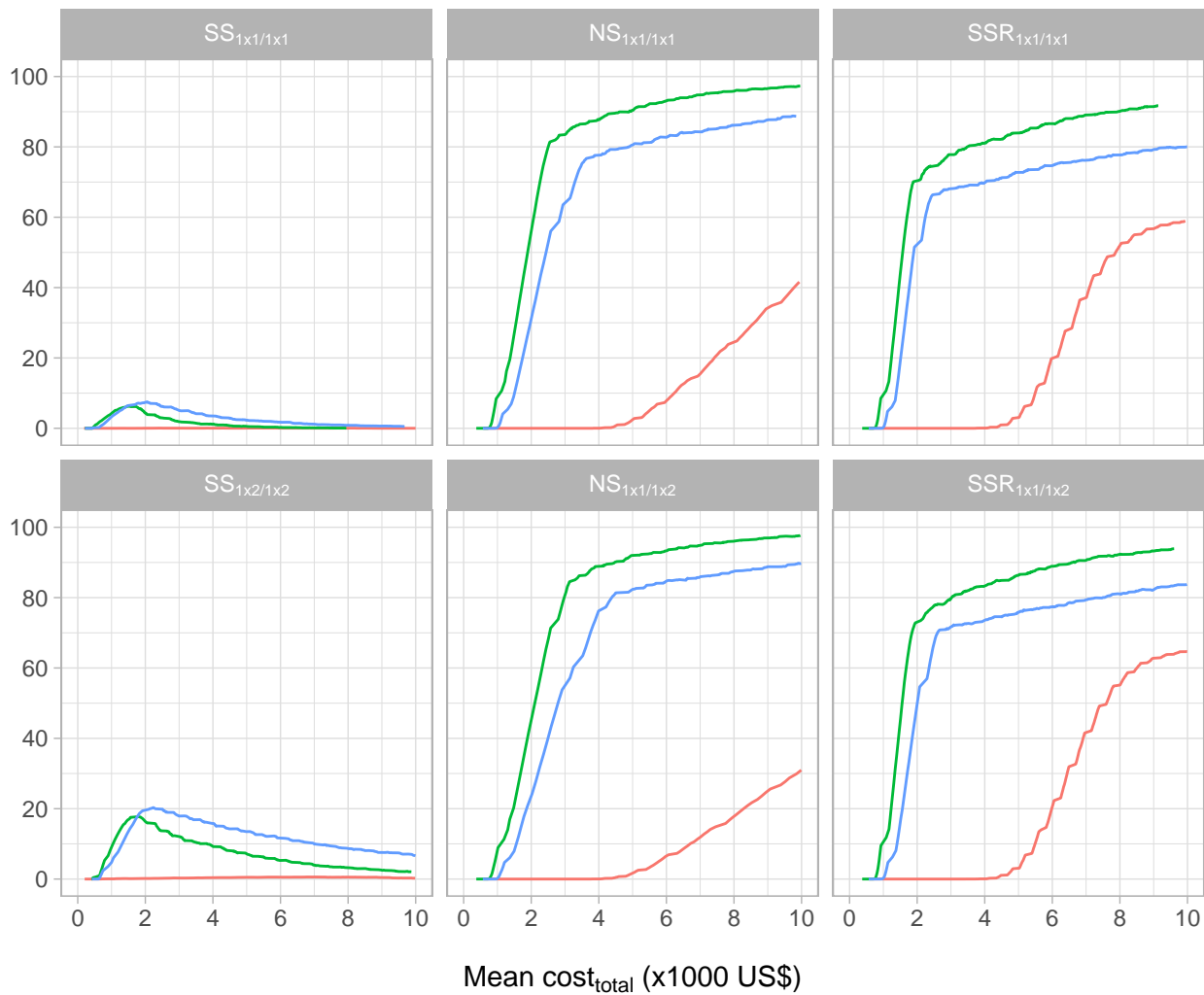

FECPAK<sup>G2</sup> KK Mini-FLOTAC

Supplement: S3 Fig — This figure plots the probability of correctly identifying reduced therapeutic efficacy (probreduced) against Ascaris infections as a function of the mean total survey costs (costtotal) for the three different FEC methods (Kato-Katz thick smear (KK), Mini-FLOTAC and FECPAKG2; colored lines) and six survey designs (different panels). For each panel, we only consider areas that are low endemic for Ascaris (mean FEC = 9.6 EPG). NS = no selection; SS = screen and select; SSR = screen, select, and retest. (PDF) [file pntd.0011071.s010.pdf]

**A**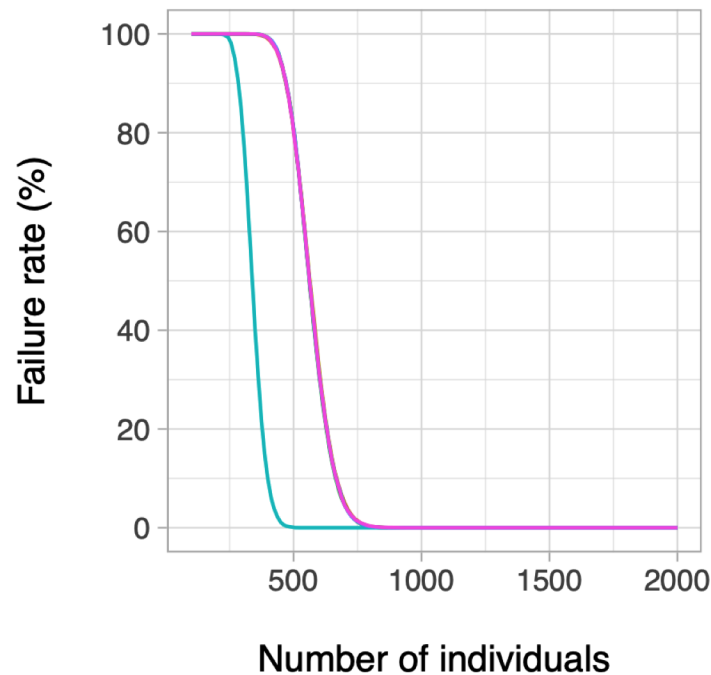**B**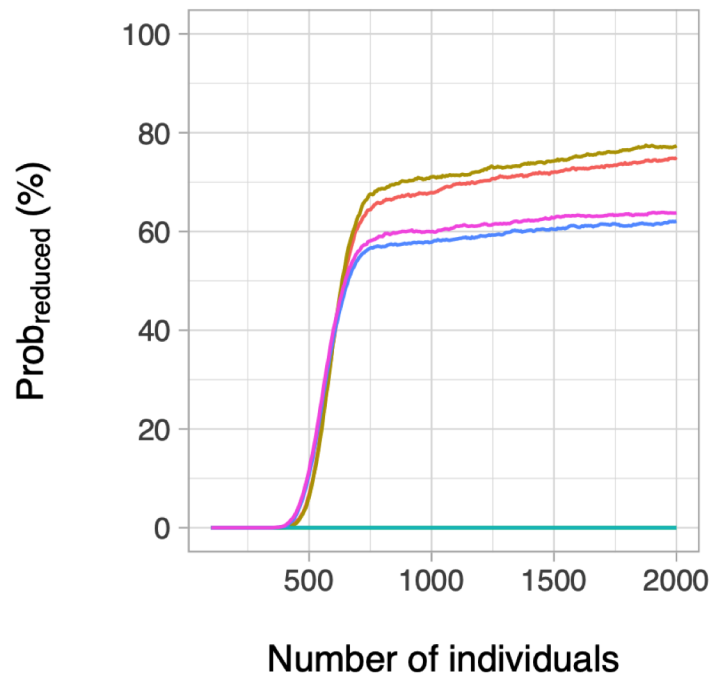**C**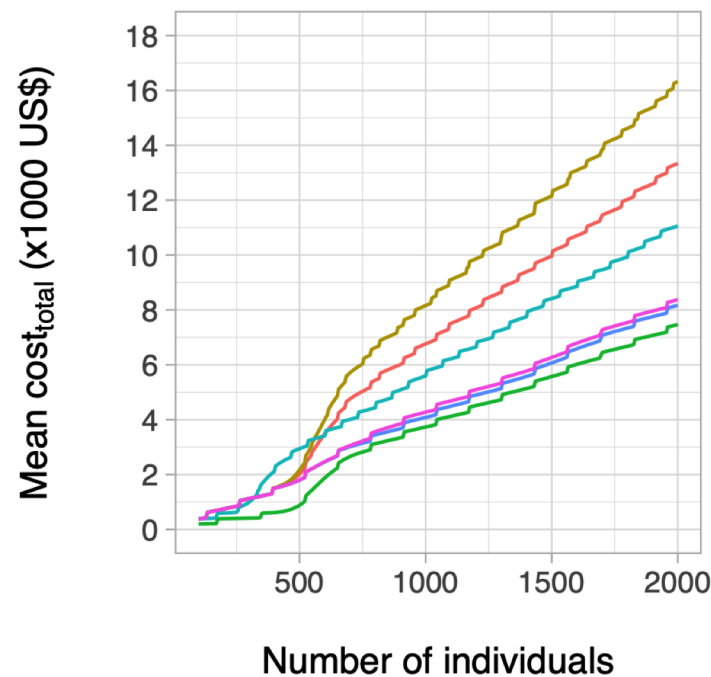**D**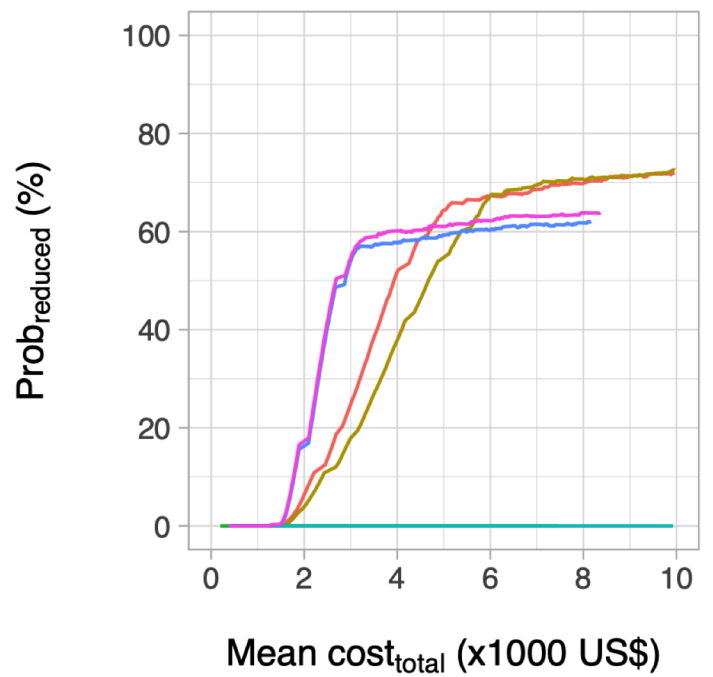

— NS<sub>1x1/1x1</sub> — SS<sub>1x1/1x1</sub> — SSR<sub>1x1/1x1</sub>  
— NS<sub>1x1/1x2</sub> — SS<sub>1x2/1x2</sub> — SSR<sub>1x1/1x2</sub>

Supplement: S4 Fig — This figure shows the impact of the survey design and sample size on the failure rate (Panel A), probability of correctly detecting truly reduced efficacy (probreduced; Panel B) and the mean total survey cost (costtotal; Panel C). To gain more insights into the most cost-efficient survey design, the probability of correctly detecting reduced drug efficacy probreduced was plotted as a function of the mean costtotal (Panel D). For each of the four panels, we only consider the use of Kato-Katz in areas with low levels of Trichuris infection (mean FEC = 2.8 EPG). NS = no selection; SS = screen and select; SSR = screen, select, and retest. Note, for panel A, all survey designs other than SS1x2/1x2 are identical to SSR1x1/1x2. (PDF) [file pntd.0011071.s011.pdf]

**A**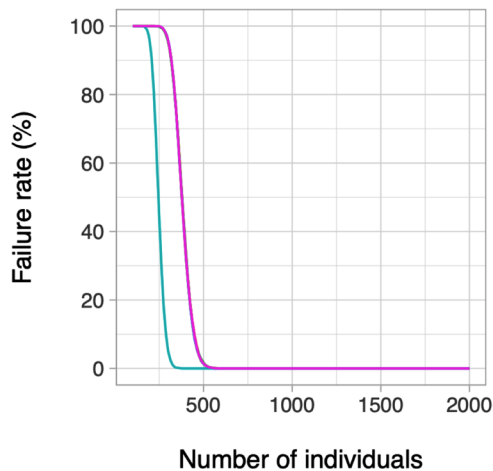**B**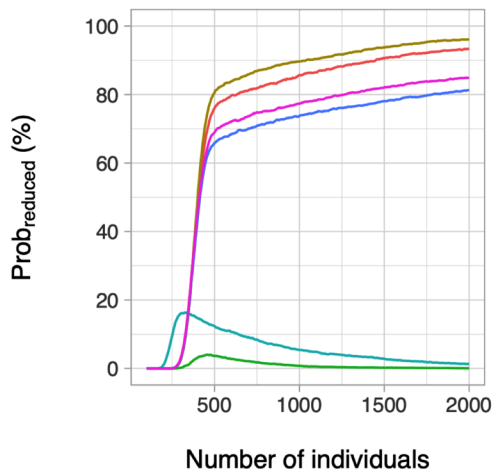**C**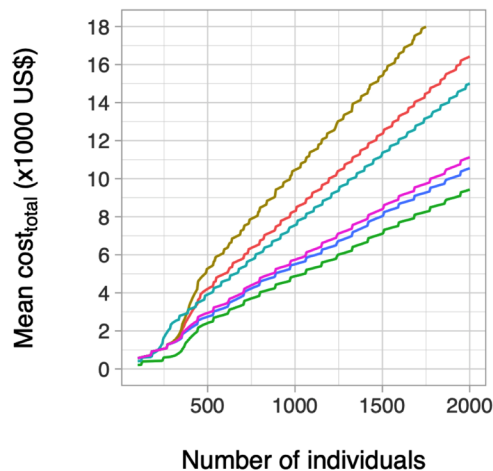**D**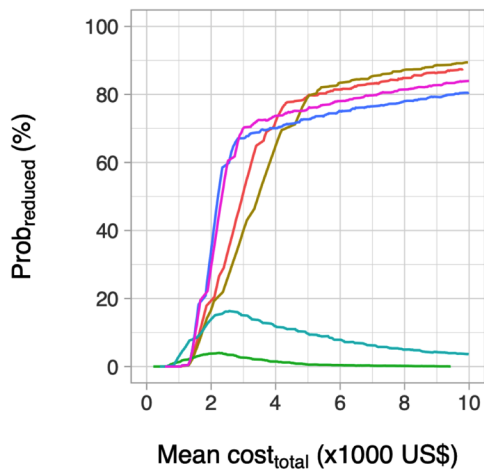

— NS<sub>1x1/1x1</sub>    — SS<sub>1x1/1x1</sub>    — SSR<sub>1x1/1x1</sub>  
— NS<sub>1x1/1x2</sub>    — SS<sub>1x2/1x2</sub>    — SSR<sub>1x1/1x2</sub>

Supplement: S6 Fig — This figure plots the probability of correctly identifying reduced therapeutic efficacy (probreduced) as a function of the mean total survey costs (costtotal) across six survey designs for the three soil-transmitted helminth species and four levels of endemicity (see Table 1). (PDF) [file pntd.0011071.s013.pdf]

**A**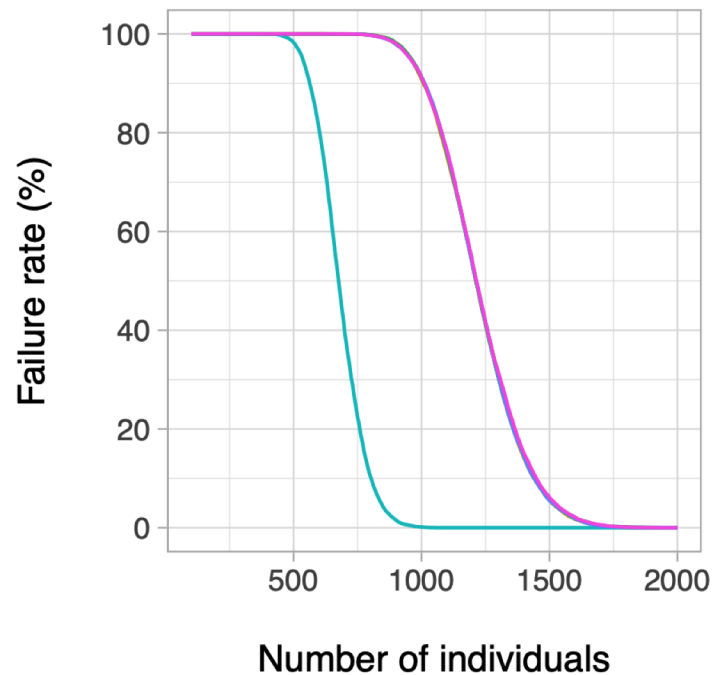**B**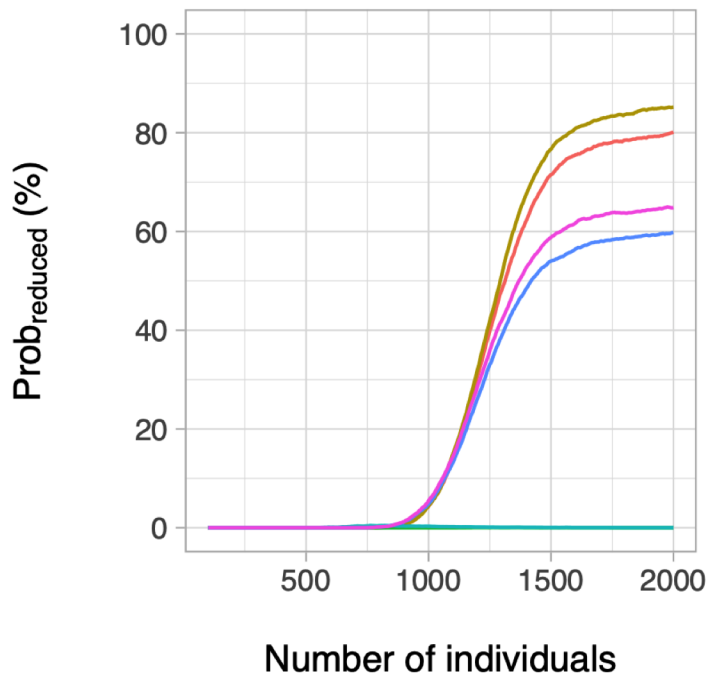**C**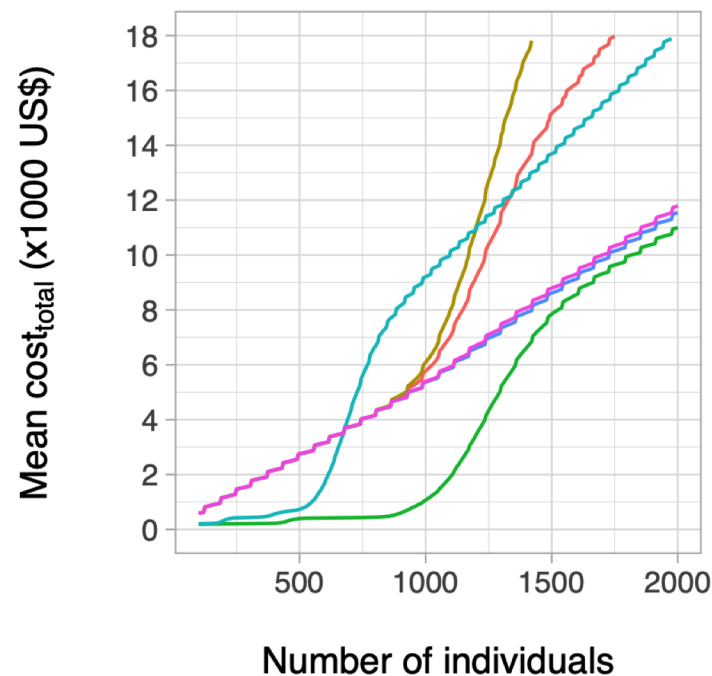**D**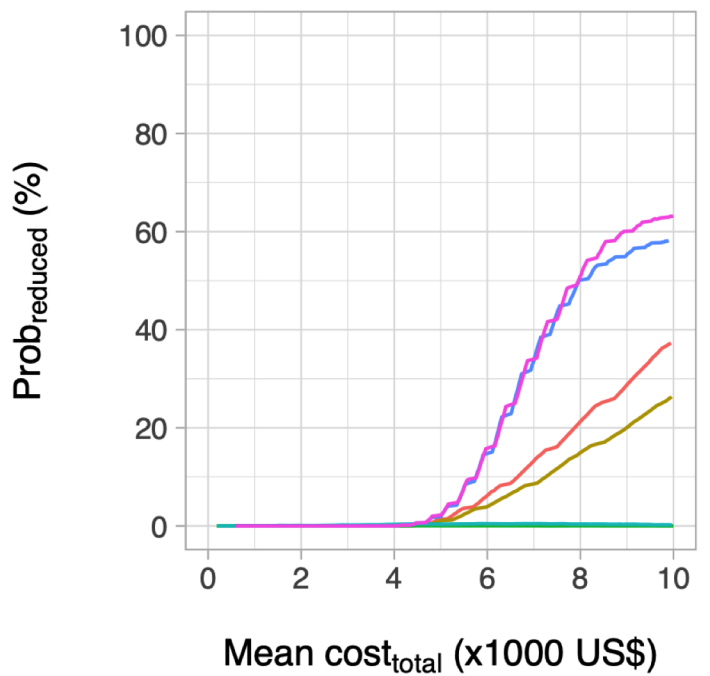

— NS<sub>1x1/1x1</sub>    — SS<sub>1x1/1x1</sub>    — SSR<sub>1x1/1x1</sub>  
— NS<sub>1x1/1x2</sub>    — SS<sub>1x2/1x2</sub>    — SSR<sub>1x1/1x2</sub>

Supplement: S7 Fig — This figure plots the probability of correctly identifying reduced therapeutic efficacy (probreduced) as a function of the mean total survey costs (costtotal) across six survey designs for the three soil-transmitted helminth species and four levels of endemicity (see Table 1). (PDF) [file pntd.0011071.s014.pdf]
