# Supplementary material for: A general framework to support cost-efficient fecal egg count methods and study design choices for large-scale STH deworming programs–monitoring of therapeutic drug efficacy as a case study
Source: PLoS Negl Trop Dis. 2023 May 17;17(5):e0011071. doi: 10.1371/journal.pntd.0011071 (PMC10228800; doi:10.1371/journal.pntd.0011071)
Supplement: S5 Fig — This figure plots the probability of correctly identifying reduced therapeutic efficacy (probreduced) against Trichuris infections as a function of the mean total survey costs (costtotal) for the three different FEC methods (Kato-Katz thick smear (KK), Mini-FLOTAC and FECPAKG2; colored lines) and six survey designs (different panels). For each panel, we only consider areas that are low endemic for Trichuris (mean FEC = 2.8 EPG). NS = no selection; SS = screen and select; SSR = screen, select, and retest. (PDF) [file pntd.0011071.s012.pdf]

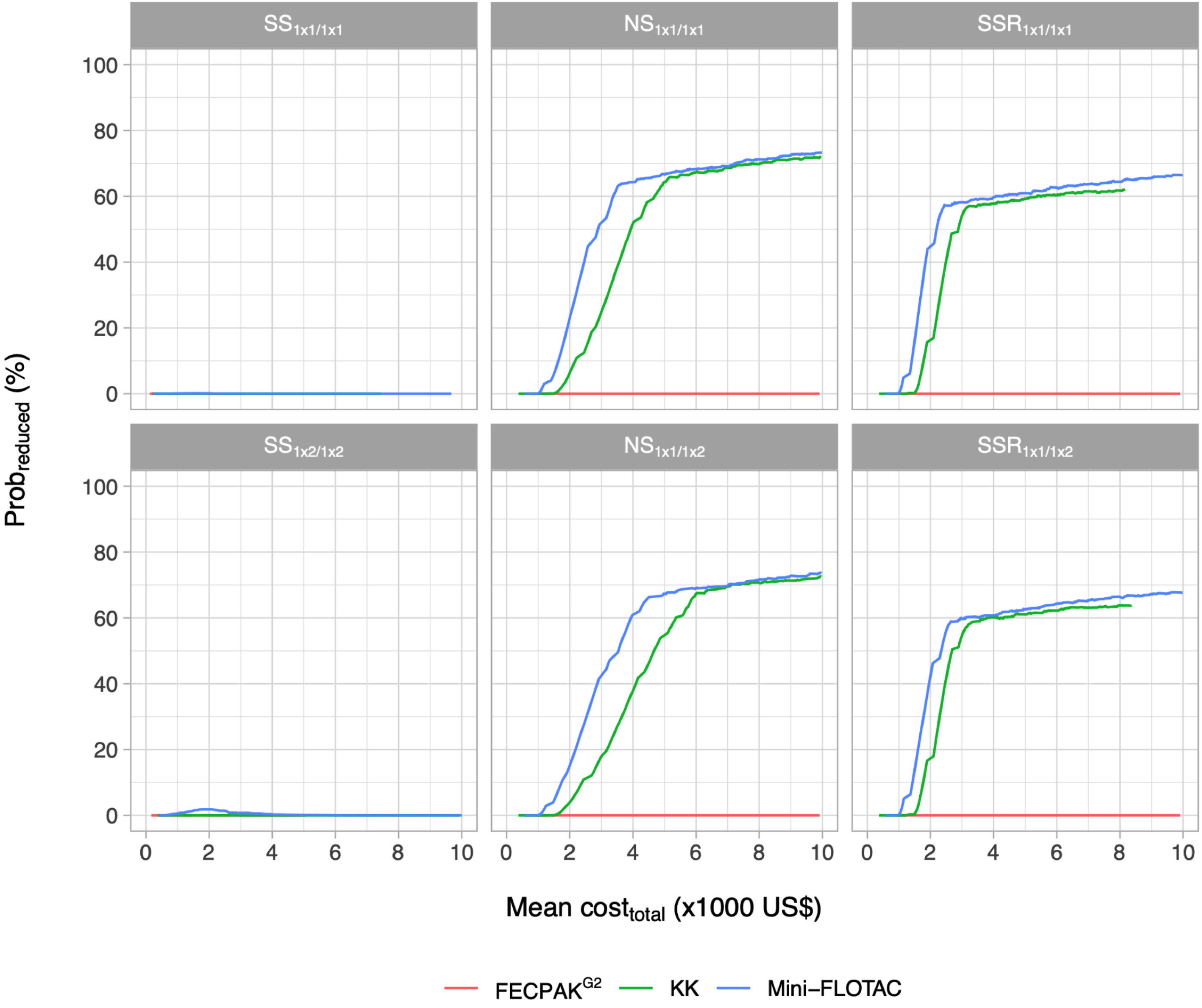

SS1x1/1x1
NS1x1/1x1
SSR1x1/1x1
SS1x2/1x2
NS1x1/1x2
SSR1x1/1x2
Probreduced (%)
Mean costtotal (x1000 US$)
0
20
40
60
80
100
0
2
4
6
8
10
FECPAKG2
KK
Mini–FLOTAC
