# Supplementary material for: A general framework to support cost-efficient fecal egg count methods and study design choices for large-scale STH deworming programs–monitoring of therapeutic drug efficacy as a case study
Source: PLoS Negl Trop Dis. 2023 May 17;17(5):e0011071. doi: 10.1371/journal.pntd.0011071 (PMC10228800; doi:10.1371/journal.pntd.0011071)
Supplement: Attachment — Submitted filename: Comments.docx [file pntd.0011071.s015.docx]

In the study "A general framework to support cost-efficient fecal egg count method and study design choices for large-scale STH deworming programs – monitoring of therapeutic drug efficacy as a case study", the authors have provided a framework for cost efficient STH survey designs for monitoring and evaluation of helminth eggs in stool samples. The authors have determined that Kato-Katz combined with a ‘no selection’ design will be most cost-efficient method. The work is original and the methodology is robust, and I recommend its publication. I only have a few comments as follows:

1. Though a detailed analysis of survey costs is included, the cost of lodging is not included. This cost is not covered in per diem cost. In most surveys, local expert technicians are not available and the teams are brought in from outside labs, so they need lodging. Depending on the sample size, the number of days of the lodging will also vary and affect the survey cost. Similarly, the to-and-fro travel cost of the team is not included. Either these costs may be included in the analysis, or mentioned in the limitations section where other such costs of salary of senior staff, etc. which are not included in the analysis are mentioned.
2. Though it would not affect the results, what is the rationale of considering a life-expectancy of 10000 samples per microscope, and 10000 entries per computer?
3. There is no mention of the number of days required for each sample size in the study design models which will depend on the sample size and the endemicity. Although the analysis of the different sample sizes and the analyses of unit costs are included, a mention of the corresponding days required for the ‘field work’ of such surveys for each sample size and design will be useful information.

the following are my comments. My comments are as follows:
