# Supplementary material for: A general framework to support cost-efficient fecal egg count methods and study design choices for large-scale STH deworming programs–monitoring of therapeutic drug efficacy as a case study
Source: PLoS Negl Trop Dis. 2023 May 17;17(5):e0011071. doi: 10.1371/journal.pntd.0011071 (PMC10228800; doi:10.1371/journal.pntd.0011071)
Supplement: Attachment — Submitted filename: Reply to reviewer comments.pdf [file pntd.0011071.s016.pdf]

Rotterdam, Thursday, 30 March 2023

Subject: submission of revised manuscript PNTD-S-23-00016

Dear Editors,

Thank you for considering our manuscript entitled "*A general framework to support cost-efficient fecal egg count methods and study design choices for large-scale STH deworming programs – monitoring of therapeutic drug efficacy as a case study"* for publication in PLoS Neglected Tropical Diseases, in the **StarWorms collection**. And thank you and the reviewers for the positive and constructive feedback.

As requested, we hereby submit a revised manuscript, addressing all the reviewer comments. We also include a list of the original reviewer comments and our replies to each individual comment (from next page onwards).

We confirm that this manuscript is not under consideration anywhere else. Thank you again for your consideration.

Sincerely,

Luc E. Coffeng, MD PhD
Department of Public Health
Erasmus MC, University Medical Center Rotterdam
The Netherlands

AUTHORS' REPLY TO REVIEWER COMMENTS (by manuscript section)

Methods

Reviewer #1

In the paragraph starting on line 184, the authors state that the stool samples were first grouped in batches of ten samples and then all samples were homogenized by stirring. It was unclear to me if the 10 samples from different individuals were mixed all together. When reading supplementary information 1, it became more clear to me that the samples were not mixed, but processed as individual samples in batches of 10. For clarity, it would be better if that was made clear in this paragraph of the main manuscript. Also, that subsamples from each of the individual samples were then processed using the three FEC methods.

Response 1

Thank you for pointing this out. We have adapted the main text as follows: *"Subsequently, ~~stool samples were~~* ***each individual stool sample was*** *homogenized by stirring with a wooden tongue depressor."*

Reviewer #2

The background and aims of the study, i.e. to provide an novel framework for cost-efficient study design to examine soil-transmitted-helminth monitoring and evaluation of deworming program efficacies. In my view the objectives were addressed appropriately and by considering key parameters including different diagnostic methods, parasite species, field-relevant different epidemiological scenarios and survey designs. The study population is clearly presented and appropriate to test the hypothesis that by comparing the above listed factors evidence-based recommendations can be developed for cost-efficient studies monitoring the efficacy of deworming programs. I don't feel competent to comment on the used statistical methods. I can't see any ethical or regulatory concerns.

Response 2

Thank you for this positive assessment!

Reviewer #4

Though a detailed analysis of survey costs is included, the cost of lodging is not included. This cost is not covered in per diem cost. In most surveys, local expert technicians are not available and the teams are brought in from outside labs, so they need lodging. Depending on the sample size, the number of days of the lodging will also vary and affect the survey cost. Similarly, the to-and-fro travel cost of the team is not included. Either these costs may be included in the analysis, or mentioned in the limitations section where other such costs of salary of senior staff, etc. which are not included in the analysis are mentioned.

Response 3

The cost of lodging is included in the personnel cost, but this was not clear from the text. We have amended in the main text: *"the ~~salary for~~* ***cost of*** *a single mobile field team comprised of one nurse and three laboratory technicians* ***(including salary and lodging)****, …"*. The description of calculation of personnel cost in **S6 Info** has also been adapted accordingly. As for to-and-fro travel cost of the team:

we now clarify in **S6 Info** that we did not include this cost as we assumed it to be the same for all survey designs, assuming that all surveys are conducted in a single location. We now also briefly touch on this in the discussion, as suggested:

"… we do not consider costs that can be reasonably assumed to be the similar across different survey designs and different FEC methods: …, ***time required for the field team to travel to and return from the study location at the start and end of the survey****, …."*

And:

"In addition, we do not consider the time required to travel to new another study location if the target sample size cannot be reached in single site, meaning that some of the larger recommended survey designs (e.g., for T. trichiura) are potentially somewhat more expensive than we estimated."

Though it would not affect the results, what is the rationale of considering a life-expectancy of 10000 samples per microscope, and 10000 entries per computer?

Response 4

This was an arbitrary choice, which, as the reviewer remarks, has little to no effect on the results. We now explain in the methods section: ***"The cost of a microscope and computer for data-entry were each amortized over 10,000 FEC samples, assuming that they both would be useable for multiple surveys"****.*

Results

Reviewer #1

The paragraph in lines 403 to 419 is confusing. Maybe some of the examples can be better displayed in table form?

Response 5

We appreciate that the level of detail in this paragraph can be overwhelming. As the paragraph is primarily intended to guide the reader through the interpretation of Fig 4, instead of adding a table, we have simplified the text considerably, while keeping the main message intact:

*"For a given sample size, the most expensive survey designs were $NS_{1\times1/1x2}$, $NS_{1\times1/1x1}$ and $SS_{1\times2/1x2}$, while the two SSR designs and $SS_{1\times1/1x1}$ were the cheapest (**Fig 4C**). For example, when enrolling 700 individuals, the mean total survey cost ($cost_{total}$) was at least 4,000 US$ for $NS_{1\times1/1x2}$, $NS_{1\times1/1x1}$ and $SS_{1\times2/1x2}$, while for the two SSR designs and $SS_{1\times1/1x1}$ the mean $cost_{total}$ was around 3,000 US$ or less. To determine the most cost-efficient survey design, we plotted the probability of detecting reduced efficacy ($prob_{reduced}$) against total survey cost ($cost_{total}$) (**Fig 4D**). For survey budgets up to 2,600 US$, the two SSR survey designs were the most cost-efficient as they maximized the probability to detect reduced drug efficacy. For budgets between 2,600 and 4,200 US$, the $SSR_{1\times1/1x2}$ design was the most cost-efficient. For budgets between 4,200 and 5,200 US$, $NS_{1\times1/1x1}$ resulted in the highest $prob_{reduced}$, whereas for a budget of 5,200 US$ or more, $NS_{1\times1/1x2}$ was the most cost-efficient survey design. To reduce the risk of falsely concluding adequate drug*

efficacy to <20% ($prob_{reduced} \geq 80\%$), $NS_{1\times1/1x1}$ was the most cost-efficient option (red line; $cost_{total}$ = 5,000 US$), with $NS_{1\times1/1x2}$ as a close runner-up (beige line; $cost_{total}$ = 5,200 US$).”

Line 445, the word "redrawing" sound like you drew the graphs instead of plotting the ascaris and trichuris data.

Response 6

We adapted the wording to: *“**When determining the most cost-efficient survey design** ~~redrawing~~ ~~**Figs 4** and **5**~~ for the other two STH species at **a** ~~the corresponding~~ low endemicity level ….”*

Reviewer #2

The study results are clearly and comprehensively presented, the figures and tables are of appropriate quality, correct and informative. The presented data match the analysis plan.

Response 7

Thank you!

Reviewer #4

There is no mention of the number of days required for each sample size in the study design models which will depend on the sample size and the endemicity. Although the analysis of the different sample sizes and the analyses of unit costs are included, a mention of the corresponding days required for the ‘field work’ of such surveys for each sample size and design will be useful information.

Response 8

Thank you for this suggestion. We have added a column to Table 4, listing the number of days required per survey.

<u>Conclusions</u>

Reviewer #2

The interpretation of the data is clear and comprehensible. The limitations (and strengths) of the study are discussed in detail in a specific paragraph of the manuscript. The whole concept of the study is to optimize the planning and conduct of deworming surveys, which also demonstrates its public health relevance.

Response 9

Thank you!

<u>Editorial suggestions</u>

Reviewer #1

In the title, the word method is missing an "s".

Response 10

Amended.

It´s not clear throughout the manuscript what is the exact range of subjects enrolled that were considered. In the abstract its states 10-2,000 (line 57), but in the method section (line 292) it says for a survey design a range of 100 - 5,000 was used.

Response 11

Thanks for spotting this. The numbers in the abstract were outdated and now reflect the numbers in the methods section.

Line 109 of the introduction, the word "those" is incorrectly used in this sentence.

Response 12

Amended.

Reviewer #2

Figure 1 with respect to the Mini-FLOTAC flow chart in my view it should not read Accumulation but Flotation.

Response 13

Thanks for spotting this. This has been amended.

Concerning the calculation of the required time to analyse a stool sample with one of the three methods I wonder why for the FECPAG method time for counting the eggs is considered? As the method is being used this is not done in the lab but online by a technician from Techion Group Ltd.? This should be explained if not also considered with respect to the time calculations.

Response 14

The FECPAK technology requires personnel time to annotate eggs in the images. In case of veterinary intestinal helminth infections, for which FECPAK is commercially available, annotation is done by the company. However, as such a service is not (yet) available for human intestinal helminth infections. We costed the time required for annotation based on the Ethiopian salaries. This probably means that if FECPAK were commercially available for human infections, FECPAK would be slightly more expensive. However, this has no repercussions for any of the findings in the paper, as FECPAK was already always outperformed.

Summary and General Comments

Reviewer #1

The article presented by the authors is relevant to the field of public health and provides useful information for STH deworming programs. Through a detailed analysis on the performance, time, and cost of three different FEC techniques they provide useful recommendations that can be readily applied. Therefore, I recommend publication of the article after a few minor revisions have been addressed.

Response 15

Thank you!

Reviewer #2

This is a very important and potentially most relevant paper for all those interested in the monitoring of STH deworming programs. I find it very clearly presented and the data novel, convincing as well as significant.

Response 16

Thank you!
